# Medical News and Items

**Published:** 1877-01

**Authors:** 


### Medical News and Items.

The *Annual Meeting* of the Stockholders of the *Chicago Medical Press Association*, was held Dec. 5, 1876, at the Grand Pacific Hotel. Drs. W. H. Byford, T. D. Fitch, and J. E. Owens were elected Directors for the ensuing three years, to fill the vacancies, by limitation, of Directorships held by Drs. Byford, Hamill and Bevan. Reports from the President, Treasurer, Secretary, Librarian and Editor, were submitted. The affairs of the Association are in as good condition as can be reasonably expected at the present time. The library contains nearly three thousand books, pamphlets, and journals. The prospects for greater increase in accumulation of medical literature are flattering. The Directors hope soon to have a complete set of the New Sydenham Society's publications, numbering about eighty volumes of very rare books, at a small outlay of money. The number of medical journals regularly received is now one hundred and seven. The list of foreign journals is continually increasing. The latest publications in the line of medical books and phamphlets are all received, and thus the library, the great objective point of the Association, is steadily increasing in worth.

The most reliable feature of this collection of medical literature is an immense Index Rerum (consisting of a large volume for nearly every letter of the alphabet), which is being written up by the Librarian, Dr. Bridge, and a corps of able assistants, selected from among the rising young physicians of Chicago. Every original communication in the journals received is indexed, and, when one considers that at least five hundred such entries are made every month in the Index, it can readily be seen what an invaluable work is steadily being prepared for the present and future generations of medical writers and students in Chicago.

Arrangements are now in contemplation, which if executed, will place the library in well lighted and warmed rooms, in charge of an Assistant Librarian, day and evening.

---

The *Tri-State Medical Society* of Illinois, Indiana and Kentucky, held its second annual meeting at Vincennes, Ind., on November 21, 22 and 23, 1876. The meetings were well attended. The following papers were read and discussed and will be incorporated in the printed transactions of the Society: On the use of Ergot, by Dr. W. H. Smith, of Newman, Ill.; The Solution and Absorption of Medicines, by Dr. J. W. Campton, of Evansville, Ind.; Report on Obstetrics, by Dr. Ireland, of Louisville, Ky.; On Treatment of Diseases of the Larynx and Vocal Cords, by Dr. B. Tauber, of Cincinnati, O.; On Nervous Diseases of the Eye, by Dr. Dickinson, of St. Louis, Mo.; Treatment of Catarrhal Affections of the Nose and Ear, by Dr. Thos. Reimbolt, of St. Louis, Mo.; Traumatic Tetanus, by Dr. Ezra Read, of Terre Haute, Ind.; The Second Decade of Life, by Dr. W. H. Byford, of Chicago; Medical Education of Europe and America, by Dr. J. O. Stittson, of Bedford,

Ind.; The Pneumonia of the Wabash Valley, by Dr. J. B. Armstrong, of Terre Haute, Ind.; Conjunctival Diseases, by Dr. J. P. Worrell, of Terre Haute; Puerperal Peritonitis, by Dr. G. W. Burton, of Mitchel, Ind.

The committee on nominations made the following report of officers for the ensuing year:

For President, Dr. W. H. Byford, Chicago, Ill.; 1st Vice President, Dr. John L. Dismukes, Mayfield, Ky.; 2d Vice President, Dr. G. G. Barton, Washington, Ia.; 3d Vice President, Dr. H. H. Deming, Pana, Ill.; Recording Secretary, Dr. G. W. Burton, Mitchel, Ind.; Corresponding Secretary, Dr. F. W. Beard, Vincennes, Ind.; Treasurer, Dr. H. Patten, Vincennes, Ind.; and the Society then adjourned to meet in the city of Evansville, Ind., on the third Tuesday in Oct., 1877.

---

The new *Cook County Hospital* has been occupied now several months. Its furnishing and appointments are nearly perfection; the beautiful wards with new furniture—and such furniture—and bright bed-spreads, and clean floors, are a sight really worth beholding; it reminds us of a dress parade with a full regiment, new clothes and white gloves. Of course the time has been too short to get everything running smoothly, but we are sorry to hear so soon complaints regarding the management. There are, we are told, six engineers in the establishment, and yet in the surgical wards in one day, the temperature is allowed to fall from 98° to 38° F., because these poor engineers must sleep. The charges of mismanagement made by patients through the daily prints of late, we forbear discussion of in this place—doubtless something may be said on both sides. The amphitheatre is expected to be ready for occupancy by the time this journal is delivered to its readers.

---

The following letter to the Physicians of the State of Maine, is self explanatory:

PORTLAND, ME., 15th of November, 1876.

DEAR SIR: At its August meeting, the attention of the

Cumberland County Medical Society was called to an alleged "Opium Antidote," which a member had found to contain morphine, and the undersigned were appointed a committee to obtain a quantitative analysis of the preparation. While this investigation was proceeding, another so-called "Opium Antidote" was brought to the notice of the committee, and was subjected to a similar examination.

The first specimen, manufactured by Mrs. J. A. Drollinger, of La Porte, Indiana, was analyzed by Walz and Stillwell, Chemists, New York City, who found it to consist of glycerine colored with aniline red and containing in solution 1.383 per cent. by weight of the sulphate of morphia — about seven grains to the ounce.

The second was the preparation of "Dr. S. B. Collins, the great Narcologist of the Age," of La Porte, Indiana. The analysis of this was made by Dr. Henry Carmichael, Professor of Chemistry in Bowdoin College and Assayer of the State of Maine, and differed from the preceding only in the amount of the sulphate of morphia shown to be present, namely, 3.2 per cent. A teaspoonful (a dose frequently prescribed by the proprietor) would contain almost two grains of the morphia — nearly twelve times the ordinary medicinal dose.

The Society instructed the Committee to publish the result of the investigations, in order that the large number of opium-eaters in this country who are earnestly striving to overcome their health-destroying habit, may be seasonably warned against these widely advertised nostrums, which, while promising a safe and sure cure, only serve to increase the appetite for opium until it finally becomes unconquerable.

This circular is sent to every physician and editor in the State of Maine, and to many other persons of prominence and influence, who will be able to give wide publicity to these facts and thus help diminish the prevalence of the opium habit, which is yearly destroying great numbers of our people. Opium eating is usually practised without the knowledge of friends, and the attempts at cure are often equally secret; therefore the information which has been acquired concerning these dangerous preparations must be extensively disseminated in order to certainly reach those for whose benefit only it was sought. Will you assist in the work by making known these facts in your neighborhood?

FREDERIC HENRY GERRISH, M.D.,

GEORGE F. FRENCH, M.D.,

THOMAS A. FOSTER, M.D.,

} *Committee.*

---

THE MICHIGAN STATE BOARD OF HEALTH.—This must be a thrifty organization. We have received an abstract of their October meeting, and notice that Dr. Kedzie presented a paper on the "Water Supply of Michigan," wherein he treated of the geological formation of the State as affecting the water supply, the mechanical and chemical effect of different kinds of soil on the water filtered through them, of the impurities in water supply, of grave yards and their sources of impurities, and the methods of improving the quality of water now used; that Dr. Hazelwood read a paper on "Water," based largely on replies of correspondents to a circular of the Board; that Dr. Baker read a paper on the "Causes of Chorea;" that Dr. B. also presented material for a paper on the "Death Rate as Influenced by Age, Climate, etc.," and that Dr. Hitchcock read a paper on "Criminal Abortion," showing that the existing laws of Michigan are based on the views of past ages, and are not in conformity with the present knowledge of physiology.

---

DR. BRUNET has recently published an interesting work on Polynesia, in which it is stated that version, by external manipulation, has been practiced by the midwives of that country from time immemorial. The vaginal touch is interdicted. The operation is performed at the onset of labor and before the rupture of the pouch of water, and is successfully accomplished. On two occasions when the doctor had convinced himself of the position by auscultation and palpation, and there was presentation of the right shoulder, the dorsum being anterior, the maneuvers of the midwives were successful

